# Child sex trafficking in the United States: Challenges for the healthcare provider

**DOI:** 10.1371/journal.pmed.1002439

**Published:** 2017-11-22

**Authors:** V. Jordan Greenbaum

**Affiliations:** Stephanie V. Blank Center for Safe and Healthy Children, Children’s Healthcare of Atlanta, Atlanta, Georgia, United States of America

## Abstract

V. Jordan Greenbaum discusses ways healthcare providers can identify children trafficked for sex to provide for their physical and mental health and their social and educational needs.

**Summary points**

- Victims of child sex trafficking are at high risk of numerous physical and behavioral health problems and are likely to seek medical attention. This places healthcare providers (HCPs) in a position to identify high-risk youth and offer critical services.
- Children are unlikely to disclose their victimization spontaneously to HCPs. To increase the likelihood that providers recognize victims and appropriately respond to their particular needs, training and resources are needed in the following 3 areas: understanding trauma and its impact on children, victim-centered and human rights–based approaches to care, and developmentally appropriate interview techniques.
- Building trust and establishing the rapport needed to allow a child victim to disclose exploitation typically requires time. This may be difficult to allocate in busy medical settings. Screening tools, division of responsibilities among staff, and prioritization of assessment for trafficking may help to address this problem.
- Trafficked children have a wide range of physical, mental health, educational, and social needs that are best met by multidisciplinary collaboration of HCPs, victim service providers, government agencies, and other stakeholders. Development of detailed hospital/clinic protocols will assist HCPs in accessing appropriate community and national resources.

## Scope and definition of child trafficking

Human trafficking violates fundamental human rights of children the world over [1,2]. In a global study by the United Nations, identified trafficked persons originated from 106 countries. Of over 17,000 victims, 28% were children, with girls outnumbering boys by a factor of 2.5[2]. According to United States federal law[3,4], sex trafficking involves “The recruitment, harboring, transportation, provision, obtaining, soliciting or patronizing of a person for the purpose of a commercial sex act (any sex act on account of which anything of value is given to or received by any person) using force, fraud, or coercion, OR involving a child less than 18 years of age.” This definition is broad relative to many countries, as it does not require transporting a victim, and it does include commercial sexual transactions between a child and another person that do not involve a third party controller (sometimes referred to as “survival sex” when applied to the homeless/runaway population). Thus, child sex trafficking (CST) includes using a minor to produce child sexual exploitation materials (“pornography”), using a child in a sex-oriented business (e.g., exotic dancing/strip club), soliciting a child for commercial sex (in person or online), and having a child perform a sex act with another person(s).

Existing data on sex trafficking victims identified in the US suggest that the vast majority are US citizens or permanent legal residents (84%) and are female (94%) [5]. However, cultural biases as well as investigative priorities likely influence the identification of victims. There is evidence that males and transgender youth are frequently involved in sex trafficking and exploitation, although they are likely underrecognized [6–9].

## Intersection of child trafficking and healthcare

Emerging evidence strongly suggests that a high percentage of child victims of sex trafficking in the US seek medical attention, and they do so in a variety of settings. In one study of confirmed and suspected victims of domestic minor sex trafficking, 80% reported seeing a medical provider within the year prior to their identification as victims. Most presented to emergency departments (63%), but a significant proportion (35%) presented to a variety of outpatient clinic settings [10]. Their health needs span both physical and behavioral health domains. CST is associated with sexually transmitted infections (STIs), HIV/AIDS, pregnancy, injuries from physical and sexual assault, post-traumatic stress disorder (PTSD), depression with suicidality, and other behavior problems [10–16]. Adolescent girls in one study had a 47% prevalence of STIs at the time of evaluation and a 32% rate of prior pregnancies [12]. Forty-seven percent of youth in another study reported suicide attempts within the past year and 78% met DSM criteria for PTSD [11]. In addition, some trafficking victims experience both sexual and labor exploitation [17,18], so they may present with health complications related to either form of trafficking.

However, trafficked children typically do not disclose their victimization [19]. Youth have fewer resources than adults and are thus less able to protect themselves from threats and violence by the trafficker. They lack the life experience and the ability to gain insight into the ways a trafficker may be manipulating them, accepting without question the trafficker’s claims that the child is at fault for their predicament or that he/she is worthless and must depend on the trafficker. Their corresponding feelings of guilt, shame, and hopelessness may prevent disclosure to HCPs. Many children have deep unmet needs that are exploited by a trafficker—the need for love, attention, a father figure, etc. A recruitment technique commonly used by traffickers is to develop a fraudulent romantic relationship with a victim, which can lead to very strong bonds, despite the presence of violence and exploitation. Children may be unable to accept the idea that their “boyfriend” is exploiting them and may protect him/her by denying exploitative acts or insisting such acts were “consensual.” Immature brain development and limited executive functioning render adolescents prone to risk-taking and seeking immediate gratification, rather than analyzing potential dangers and weighing options [20]. Finally, youth may not disclose their exploitation because health professionals do not ask questions.

Very young children may be victims of sex trafficking, especially in the form of prostitution or production of child sexual abuse materials. They may lack the verbal skills to disclose and the social maturity to understand their exploitation. If they are aware of their victimization and are traumatized by it, their symptoms of stress may be nonspecific and misinterpreted by others (tantrums, anxiety, sleep problems)[21]. Thus, caregivers and HCPs may remain unaware of the exploitation.

## Challenges for the healthcare professional

### Recognition of high-risk patients

To assist child victims of sex trafficking, HCPs must be able to recognize them in a busy medical setting, in which a disclosure is unlikely. Practitioners face challenges in knowing what questions to ask and potential indicators to observe and in setting aside the time needed to ask those questions in a sensitive, trauma-informed, victim-centered manner. They must keep in mind the possibility that the child’s parents may be 1) victims of human trafficking, themselves, 2) the persons trafficking the child, or 3) not the actual parents.

Knowledge of human trafficking is generally lacking among HCPs; in one study, 63% reported that they had never received training on how to identify sex trafficking victims [22]. Knowledge of risk factors for exploitation may allow the HCP to identify at-risk youth while obtaining a medical and social history (see Table 1). Fortunately, there is a recent trend toward educating providers about human trafficking [23,24] and evidence to suggest it may result in increased knowledge and awareness [25]. National medical societies, including the American Academy of Pediatrics, are publishing guidelines on the recognition and response to human trafficking [19] or issuing statements and policies calling for training of HCPs [26–28]. Medical institutions are beginning to develop specific human trafficking protocols to guide professionals through the process of recognition, evaluation, referral, and service provision [29]. Numerous resources are available from private and governmental agencies, many directed to HCPs (National Human Trafficking Resource Center: traffickingresourcecenter.org; U.S. Department of Health and Human Services, SOAR to Health and Wellness Training: https://www.acf.hhs.gov/otip/training/soar-to-health-and-wellness-training; HEAL Trafficking: https://healtrafficking.org/).

**Table 1:** Risk factors for child trafficking [9–12,30–33].

Child Factors	Family Factors	Community Factors	Societal Factors
Abuse/neglect	Poverty	Natural disaster	Gender bias/discrimination
Substance misuse	Family violence	Social upheaval	Poor acknowledgment of children’s rights
Untreated mental health/behavioral health issues	Family dysfunction	Tolerance of exploitation	Sexualization and objectification of females
LGBTQ status	Migration	Lack of educational and job opportunities	Systemic inequalities
Runaway/thrownaway*/ homeless or unaccompanied		Violence	
Involvement with juvenile justice or child protective services		Lack of awareness of trafficking	
Race/ethnicity			

**Abbreviations:** LGBTQ, Lesbian, gay, bisexual, transgender, queer/questioning.

*Thrownaway: refers to a child who has been told to leave the home or told not to come back.

### Trauma-informed care

Knowledge of common risk factors and possible indicators of child trafficking informs the HCP of appropriate questions to consider, but this knowledge alone is insufficient to adequately identify and assist potential victims. Questions must be asked in a trauma-informed, culturally sensitive, victim-centered manner [34]. This “trauma-informed approach” requires skills not usually taught in health professional programs or practiced in busy healthcare settings. The common assumptions made by HCPs that the patient is telling the truth to the best of their ability, accurately describing their condition, interested in receiving assistance from the provider, and trusting of the medical staff may prove unjustified when interacting with child trafficking victims. The provider needs to build trust, assume a nonjudgmental attitude, convey respect for the patient, ensure a sense of safety, and empower the youth to participate in the evaluation and decisions about referrals and treatment. The HCP needs to understand the impact of trauma on the patient’s views of self and others, on their behavior, their attitudes, and even their choice of words when communicating with others [35,36]. It is critical to realize that a patient’s guarded manner, belligerence, aggression, or withdrawal may be manifestations of traumatic stress and represent adaptive behaviors the child has developed to survive in their hostile environment. Meeting bellicosity with equanimity rather than sarcasm may be difficult unless the provider is aware of the dynamics and manifestations of trauma.

Increasingly, trauma-informed care is being emphasized in training of HCPs, especially in modules covering human trafficking and intimate partner violence. Numerous resources are available (National Child Traumatic Stress Network: www.nctsn.org; Children’s Healthcare of Atlanta: https://www.choa.org/csecwebinars; Polaris: https://humantraffickinghotline.org/; National Health Collaborative on Violence and Abuse: http://nhcva.org/2014/04/15/webinar-human-trafficking/; Christian Medical and Dental Associations: https://cmda.org/resources/publication/human-trafficking-continuing-education). However, there is a need for systematic and widespread dissemination of these resources. The trauma-informed paradigm of patient care needs to be taught early in the career of HCPs, as this will optimize communication with all patients, even if the provider is not aware of their trauma history.

Formal, videotaped forensic interviews of children suspected of being sexually abused or exploited are considered standard of care in many areas of the US [37], and these interviews are conducted by professionals specifically trained in child development and techniques for gaining information in an objective, legally defensible manner [38,39]. Typically, medical practitioners need to minimize questions about exploitation and ask only basic questions that help determine risk and inform strategies for exam, testing, treatment, and referrals. However, most HCPs lack training on optimal strategies of obtaining accurate information and may be unaware that open-ended questions inviting free narrative (e.g., “tell me everything you remember about…”) are preferable to yes/no questions, leading questions (“How often did he beat you?” when child has not disclosed any violence), or suggestive questions (“You told him to stop, didn’t you?”) [40]. Training in techniques for talking with children and adolescents would be helpful to HCPs and may be incorporated into medical and nursing school curricula.

### Time needed to build rapport and complete the assessment

Time is arguably one of the greatest barriers to HCP intervention in human trafficking. Creating time to build rapport and establish trust in a busy clinical setting is difficult. However, practitioners always make time for the acutely injured youth who arrives unannounced in the emergency department, for the acute sexual assault victim, for the actively suicidal child. No matter how busy the setting, time is made for situations in which danger is present and a child’s wellbeing is in jeopardy. This commitment by the medical profession needs to extend to children at risk for sex trafficking. These children are in danger, they are at great risk of future harm in the absence of intervention, and they are in need of attention. Just as healthcare professionals make time for emergency surgeries, they need to make time to talk to their at-risk youth.

The responsibility for assessing possible sex trafficking and providing referrals need not always fall to the physician. Having a designated, trauma-trained professional such as a nurse or social worker to interview potential trafficked persons, offer resources, and make necessary reports and referrals may be an efficient way to manage clinic/hospital demands. Alternatively, self-administered patient screens may be introduced to identify high-risk patients so that resources can be directed appropriately. Having patients complete questionnaires in the waiting area or exam room decreases the demand on staff resources, although one must carefully consider the circumstances under which patients may be completing the assessment. Safety and/or confidentiality may be compromised if the patient is accompanied by a trafficker or someone working for the trafficker or if a child is in the company of a parent. It would be very important to ensure that the patient has the opportunity to complete the questionnaire when outside the presence of any accompanying person.

Screening a child for possible commercial sexual exploitation assumes the existence of clinically validated screening tools that are appropriate for busy medical settings. Currently, multiple tools are available or being developed, but clinical validation is lacking in most [12,41]. It will be important to create such tools and determine factors that increase or decrease the likelihood of disclosure. Research is needed to inform us of the best way to assess children of different ages (written versus verbal versus web-based questions, the appropriate time to conduct the assessment during the visit, etc.).

### Complexity of victim needs

Trafficked children and youth have many unmet needs that extend well beyond physical and emotional health, including shelter, food, immigration and other legal assistance, language classes, education/job skills training, life skills training, and other services [19,42,43]. The complexity of patient circumstances requires a multidisciplinary approach to investigation and service provision. Such an approach is not new and much can be learned from examining the practices in the fields of child maltreatment, immigrant/refugee health, and intimate partner violence. The gold standard for child abuse assessment and intervention involves a multidisciplinary team that includes law enforcement, child protective services, mental health professionals, medical professionals, school personnel, and public health professionals [44]. There is a move to use these same mechanisms for multidisciplinary care for trafficked youth by referring them to child advocacy centers for comprehensive assessment and care.

Most pediatric HCPs have not worked extensively with nonmedical partners and may have little knowledge of what to do or who to call. Detailed protocols for clinic/hospital settings may assist HCPs in responding appropriately and providing critical resources [29], although clinical validation of such protocols is needed. They may delegate responsibilities to healthcare professionals with relevant experience such as sexual assault nurse examiners/sexual assault response teams (SANEs/SARTs) or hospital social workers, who are trained to interact with outside organizations and agencies. The protocols should maintain an up-to-date listing of community, state, and national resources, including the **National Human Trafficking Resource Center contact information (1-888-3737-888).** Adequate description of safety measures is important, as well, to ensure staff and patients are protected from harm.

## Conclusions

Healthcare professionals face a number of challenges in fulfilling their roles of identifying and assisting victims of CST. Many challenges stem from a lack of awareness and training, and efforts are underway to provide critical information and resources to HCPs. Guidelines have been published for providers and numerous curricula designed for those who may encounter trafficking victims. Some of this training is generalizable to pediatric care of all kinds and is best addressed at the level of basic healthcare professional training (e.g., medical and nursing schools). The challenge of allocating the time needed to adequately assess and serve high-risk patients is a major one and may be addressed through hospital/clinic protocols, division of staff responsibilities, patient screening tools, and a commitment to prioritize these patients in a busy healthcare setting. Protocols may also assist HCPs in working with outside agencies and organizations to help provide for the complex needs of trafficked children.

## Abbreviations

CST: child sex trafficking
HCP: healthcare provider
HIV/AIDS: human immunodeficiency virus/acquired immune deficiency syndrome
PTSD: post-traumatic stress disorder
SANE/SART: sexual assault nurse examiner/sexual assault response team
STI: sexually transmitted infection
